# PATTERNS OF RUSHED AND UNFINISHED CARE AMONG CARE AIDES IN NURSING HOMES: A LATENT CLASS ANALYSIS

**DOI:** 10.1093/geroni/igae098.1897

**Published:** 2024-12-31

**Authors:** Cybele Angel, Yuting Song, Yinfei Duan, Carole Estabrooks

**Affiliations:** University of Alberta, Edmonton, Alberta, Canada; Qingdao University, Qingdao, Shandong, China (People’s Republic); University of Alberta, Edmonton, Alberta, Canada; University of Alberta, Edmonton, Alberta, Canada

## Abstract

Rushed care and unfinished care (also called missed care, care rationing) are common in nursing homes. Although the two phenomena are interrelated, most researchers have studied them separately and used a variable-centered approach. We took a person-focused approach and aimed to identify groups of care aides who rushed care and left care tasks unfinished in similar ways. This cross-sectional analysis used survey data from 3546 care aides working in a random sample of 87 urban nursing homes collected from September 2019 to February 2020 in three provinces in western Canada. We presented to care aides a list of physical and social care tasks (e.g., bathing, talking with residents). They answered whether or not they rushed or left a care task unfinished in their previous shift of work. We performed latent class analysis to identify patterns of rushed and unfinished care and verified the identified patterns with a different wave of data. A 4-class model emerged as the best-fit model for our data. Group 1 reported high levels of rushed and unfinished care, and Group 2 was low on both rushed and unfinished care. Group 3 reported high rushed care and Group 4 reported medium rushed care; both groups reported low unfinished physical care and medium unfinished social care. Our findings suggest the complexity of rushed and unfinished care among care aides. System-level support for this workforce is urgently needed to develop targeted interventions for sub-groups of care aides to reduce rushed and unfinished care in nursing homes.

